# Accuracy and reproducibility of a retrospective outcome assessment for lumbar spinal stenosis surgery

**DOI:** 10.1186/1471-2474-13-83

**Published:** 2012-05-29

**Authors:** Pekka Kuittinen, Timo Juhani Aalto, Tapani Heikkilä, Ville Leinonen, Sakari Savolainen, Petri Sipola, Heikki Kröger, Veli Turunen, Olavi Airaksinen

**Affiliations:** 1 Department of Neurosurgery, Kuopio University Hospital, Kuopio, Finland; 2 Institute of Clinical Medicine, University of Eastern Finland, Kuopio, Finland; 3 Kyyhkylä Rehabilitation Center and Hospital, Mikkeli, Finland; 4 Department of Physical and Rehabilitation Medicine, Kuopio University Hospital, Kuopio, Finland; 5 Department of Neurosurgery, Kuopio University Hospital, Kuopio, Finland; 6 Department of Clinical Radiology, University of Eastern Finland and Kuopio University Hospital, Kuopio, Finland; 7 Department of Orthopaedics and Traumatology, Kuopio University Hospital and Bone and Cartilage Research Unit, University of Eastern Finland, Kuopio, Finland; 8 Department of Orthopaedics and Traumatology, Kuopio University Hospital, Kuopio, Finland

**Keywords:** Lumbar spinal stenosis, Surgical treatment, Outcome measures

## Abstract

**Background:**

Retrospective assessment of surgery outcome is considered problematic. The aims of this study were to evaluate the reproducibility and accuracy of a retrospective outcome assessment of lumbar spinal stenosis surgery with reference to prospective outcome scale measurements.

**Method:**

Outcome of surgery from 100 lumbar spinal stenosis (LSS) patients was evaluated retrospectively from patient files of a 3-month outpatient visit performed according to a standard clinical protocol by two independent researchers. In the retrospective analysis, outcome was graded as 2 = good if the clinical condition had clearly improved, 1 = moderate if it had just slightly improved, 0 = poor if it had not improved or was even worse than before the surgical treatment (Retrospective 3- point scale). A prospectively assessed Oswestry Disability Index questionnaire (ODI), Visual analogue pain scale (VAS) and a patient satisfaction questionnaire were used as references of standards. Reproducibility of the measurements was evaluated.

**Results:**

The retrospective 3-point scale correlated with ODI (r = 0.528; P < 0.001) and VAS (r = 0.368; P < 0.001). The agreement was better in the good and poor outcome than in the moderate outcome. Retrospective 3-point scale demonstrated substantial intra-rater and inter-rater repeatability (κ = 0.682, P < 0.001 and κ = 0.630, P < 0.001, respectively).

**Conclusions:**

Retrospective assessment of spinal surgery outcome is highly reproducible. Accuracy is highest in the patients with poor and good surgical result.

## Background

Lumbar spinal stenosis (LSS) is the most common indication for lumbar spinal surgery in people aged over 65 years [1]. The long-term results of surgery are poor in one third of patients [1,2], emphasizing the need for investigation of the predictive factors of surgical outcome [2,3] and patient selection for surgery [4]. Prospective studies are the best way to perform research. In prospective studies, however, patient selection may differ from the patient selection in daily clinical routine. In addition, comparison of treatment with historical controls is not feasible. Retrospective studies can include large patient materials. However, assessment of outcome in retrospective analysis is questionable. To the best of our knowledge, however, no previous study has investigated the accuracy and reproducibility of retrospective outcome measurements. Accordingly, the aims of this study were to evaluate the reproducibility and accuracy of a retrospective outcome assessment for lumbar spinal stenosis surgery with reference to prospective outcome scale measurements. As a model cohort we used a well characterized patient cohort which has undergone surgery for lumbar spinal spinal stenosis in a prospective study design.

## Methods

### Patients

The study included 102 patients with both clinically and radiologically defined lumbar spinal spinal stenosis (LSS) who had been selected for surgical treatment. The collection of the study cohort has been described in detail previously [5,6]. Briefly, selection for surgery was made by an orthopaedist or neurosurgeon between October 2001 and October 2004 in Kuopio University Hospital, Kuopio, Finland. The inclusion criteria were: (1) presence of severe back, buttock, and/or lower extremity pain with radiographic (computed tomography, magnetic resonance imagining, myelography) evidence of compression of the cauda equina or exiting nerve roots by degenerative changes (ligamentum flavum, facet joints, osteophytes and/or disc material), and (2) the surgeon’s clinical evaluation that the patient had degenerative LSS that could be treated operatively. In addition, all patients had a history of ineffective response to conservative treatment over three months. At the 3-month follow-up, two of the 102 baseline patients had missing BDI and ODI data, thus the final sample size was 100.

The exclusion criteria were: emergency or urgent spinal operation precluding recruitment and protocol investigations; cognitive impairment prohibiting completion of the questionnaires or other failures in co- operation; and the presence of metallic particles in the body preventing the MRI investigation. The surgeons sent the information of eligible patients to the Department of Physical and Rehabilitation Medicine, which organized the study. A previous spine operation or co-existing disc herniation (N = 13) were not exclusion criteria. Sixteen patients (out of 100 study patients) had previously undergone one or more lumbar spine operations. Seventeen patients had only lateral spinal stenosis.

All the 100 patients had open or microscopic decompressive surgery with (N = 19) or without (N = 81) arthrodesis or with extirpation of disc herniation (N = 7). Decompressive surgery included laminotomy, hemilaminectomy or laminectomy with undercutting facetectomy. Decompression was done at 1 level in 23 patients, 2 levels in 51 patients, 3 levels in 24 patients and 4 levels in 2 patients. The most common level for decompression was L4-L5. Of the 19 cases with concomitant degenerative spondylolistesis leading to posterolateral fusion, three reached two levels, and the remaining 16 cases were single level.

The study was approved by the Ethics Committee of Kuopio University Hospital, and the patients provided informed consent.

### Retrospective outcome scale measurement

In the retrospective analysis, surgical outcome was evaluated from the medical records by two independent researchers blinded for the prospective questionnaire data. Patient outcome was graded as 2 = good if the clinical condition had clearly improved which was the case when the patient was satisfied to the surgical treatment and symptom free, 1 = moderate if it had only slightly improved symptoms and the patient was not fully satisfied to the surgical treatment, 0 = poor if it had not improved symptoms or was worse than before the surgical treatment which was the case if the patient was totally dissatisfied to the surgical treatment (Retrospective 3-point scale). The judgement was based on the information in the medical records during the postoperative 3-month clinical check-up when the surgeon met the patient and patient told for the surgeon about how he or she was doing and how satisfied patient was to surgical treatment. To assess the inter-rater repeatability of the retrospective scale, the evaluation of the patient files was repeated completely for all patients (N = 100) by an independent senior neurosurgeon blinded for the previous evaluation. To assess the intrarater repeatability, the retrospective evaluation of the patient files was repeated completely (N = 100) of at least 2 months after the first evaluation by the first independent researcher, who was again blinded for previous results and prospective questionnaire data.

### Prospective outcome scale measurements

Overall back and leg pain intensity was assessed by a self-administered Visual analogue scale (VAS) (range 0–100 mm). This has been proved to be a valid index of experimental, clinical and chronic pain [7]. Subjective disability was measured by the validated Finnish version of the Oswestry Disability Index, where 0 % represents no disability and 100 % extreme debilitating disability [8,9]. Depression was assessed with the Finnish version of the 21-item BDI with scores ranging from 0 to 63 [10,11]. Patients completed the ODI, VAS and BDI questionnaires at the baseline and 3 month after operation.

### Statistical analyses

Associations between the retrospective 3-point surgical outcome scale and the prospectively measured (baseline, 3-month follow-up and change) ODI, VAS and BDI were analysed using Spearman correlation coefficients. We analysed separately analysis for patients with the only isolated lateral spinal stenosis to study possible difference outcomes in the central and lateral spinal stenosis patients. The inter-rater and intra-rater repeatability of the retrospective scale was analysed by calculating kappa coefficients (κ). Statistical significance was set at the P < 0.05 level.

## Results

**Figure 1:**
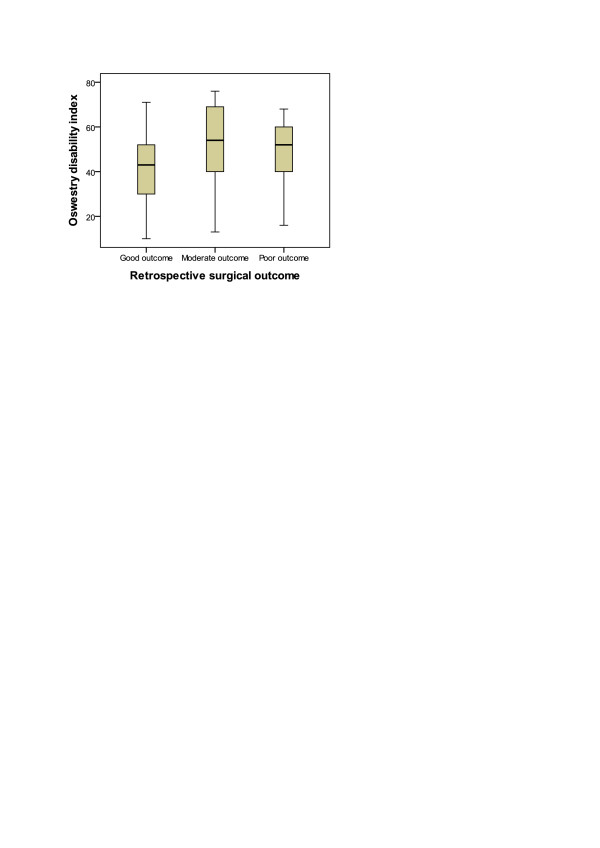
Correlation of retrospective 3-point surgical outcome and 3-month follow-up prospective Oswestry disability index.

The mean age of the study patients at the time of surgery was 62 years (range 34–86), and 57 (57 %) were male. The mean 3-month ODI was 26.9 (SD = 18.6), the mean 3-month VAS was 19.1 (SD = 22.1), and the mean 3-month BDI was 8.0 (SD = 5.8). Other background and baseline clinical characteristics of all the surgically treated lumbar spinal stenosis patients are in Table 1. According to the 3-point retrospective outcome scale, 73 (73 %) patients had good, 14 (14 %) moderate and 13 (13 %) poor outcome. 3-point retrospective outcome scale correlated with The mean 3-month ODI (Spearman r = 0.528; P < 0.001) (Table 2) (Figure 1). Spearman correlation coefficient was somewhat higher in patients with lateral canal stenosis only (r = 0.621, P = 0.008, N = 17) than in patients with central canal stenosis (r = 0.520, P = 0.001, N = 83).

**Table 1:** Background and clinical characteristics of the lumbar spinal stenosis patients preoperatively and on 3-month postoperative follow-up time n = 100

	Preoperative phase	3-months follow-up
Age (years at operation, mean (SD))	62.0 (12.0)	
Male/Female (n)	57/43	
BMI (kg/m^2^) (SD)	29.4 (4.0)	
Marital status (%)	64.4	
In relationship (married or co-habiting)		
Employment status (%), at work	13.9	
Current smoker (%)	20.6	
Number of somatic diseases (mean (SD))	5.4 (3.2)	
Type of stenosis central/lateral	83/17	
Dural sac area (mean; mm^2^) the most stenotic level	57.8 (32.5)	
Previous lumbar operation (n)	16	
Time since first back pain episode, years (mean (SD))	15.8 13.9)	
Oswestry (ODI) % (mean (SD))	43.9 (15.4)	26.9 (18.6)
VAS, mm (mean (SD))	33.3 (23.9)	19.1 (22.1)
BDI score (mean (SD))	10.3 (6.0)	8.0 (5.8)
Walking capacity, metres (mean (SD))	594 (439)	

*ODI* = Oswestry disability index scale (0-100)

*VAS overall* = Visual analogue pain scale (0-100)

*BDI* = Beck Depression index (0-63)

**Table 2:** Correlation of retrospective 3-point surgical outcome and prospective follow-up measures (N =100)

	3-month follow-up
ODI	r = 0.528, P = 0.000
BDI	r = 0.300, P = 0.002
VAS	r = 0.368, P = 0.000

*P* = P values

*r* = Spearman correlation coefficients

*ODI* = Oswestry disability index

*BDI* = Beck depression index

*VAS* = Visual analogue pain scale

**Figure 2:**
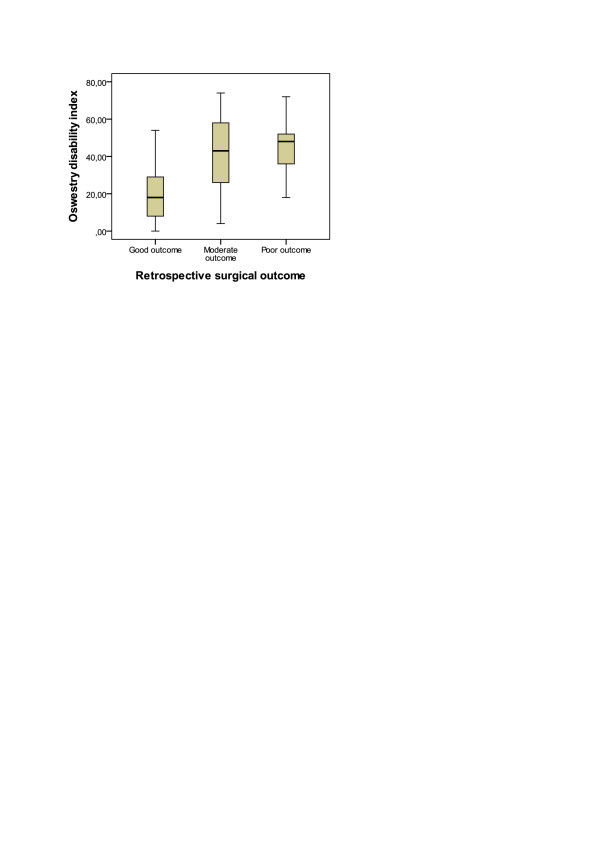
Correlation of retrospective 3-point surgical outcome and baseline prospective Oswestry disability index.

**Figure 3:**
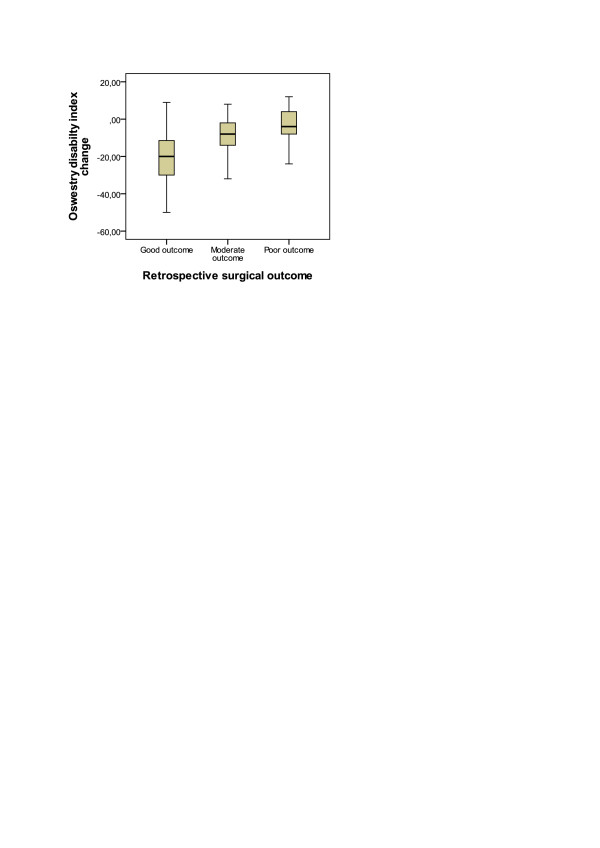
Correlation of retrospective 3-point surgical outcome and change between the baseline and 3-month follow up time prospective Oswestry disability index.

3-point retrospective outcome scale and it correlated with the mean 3-month VAS (Spearman r = 0.368, P < 0.001) (Table 2). Spearman correlation coefficient was higher in patients with lateral canal stenosis only (r = 0.592, P = 0.012, N = 17) than in patients with central canal stenosis (r = 0.335, P = 0.002, N = 83). 3-point retrospective outcome scale correlated with the mean 3-month BDI (Spearman r = 0.300, P < 0.005) (Table 2). Spearman correlation coefficient was again higher in patients with lateral canal stenosis only (r = 0.655, P = 0.004, N = 17) than in patients with central canal stenosis (r = 0.229, P = 0.038, N = 83). 3-point retrospective outcome scale correlated with the baseline ODI (r = 0.229, p = 0.022) (Figure 2), VAS (r = 0.197, p = 0.049), BDI (r = 0.292, p = 0.004) and with the change between the baseline and 3-month follow-up ODI (r = 0.482, p = 0.000) (Figure 3) but not with the change in VAS (r = 0.165, p = 0.102) and BDI (r = 0.051, p = 0.621).

We did not find any statistically significant difference when comparing the baseline and follow-up ODI, VAS and BDI scores or their change between the patients with pure spinal stenosis to those with concomitant instability or with concomitant disc herniation (Table 3).

**Table 3:** Mean (SD) change between the baseline and 3 month follow-up ODI, VAS and BDI scores

	ODI	VAS	BDI
spinal stenosis with disc herniation (n = 7)	16.0 (SD)	24.6 (SD)	4.6 (SD)
spinal stenosis with instability (n = 19)	12.1 (SD)	27.8 (SD)	23.7 (SD)
distinct spinal stenosis (n = 74)	15.5 (SD)	23.7 (SD)	4.1 (SD)
P-value	0.899	0.220	0.263

*ODI* = Oswestry disability index

*BDI* = Beck depression index

*VAS* = Visual analogue pain scale

Both the intra and inter-rater repeatability of the retrospective 3-point surgical outcome scale was substantial (κ = 0.682, P < 0.001 and κ = 0.630, P < 0.001, respectively). Overall agreement was 83 % (N = 68) and there was only one case with total disagreement in the surgical result between the researchers.

## Discussion

Selection of patients for surgical treatment of LSS still remains challenging as well as the evaluation of the efficacy of the treatment. The definition of the outcome by different outcome measures of surgical and non-surgical treatment requires clarification. To the best of our knowledge, there are no previous studies validating the retrospective evaluation of surgical outcome for lumbar spinal stenosis. Such a measure is important when studying large cohorts of patients and comparing prospective registries with previous clinical results.

In prospective studies, the outcome of treatment can be measured with standard questionnaires such as the Oswestry Disability Index (ODI) [8] and the Roland-Morris Disability Questionnaire (RDQ) [12], the Visual analogue pain scale (VAS) [13], the work disability time [14,15] and quality of life questionnaires such as SF-36[16], EQ-5D[17] and 15D [18]. Comorbidity measures such as the Beck Depression Index (BDI) [10] and the Fear-Avoidance Belief Questionnaire (FABQ) [19] are also used.

Our results show that the outcome of surgery can be evaluated also retrospectively. Accuracy is highest in patients with poor and good surgical result. Both the intra- and also the inter-rater reproducibility of retrospective assessments are acceptable. The moderate outcome is the most challenging to determine and its retrospective evaluation could be questioned (Figure 1).

This study indicates that patients who had at the baseline worse scores in the ODI, VAS and BDI had also worse surgical outcome according the retrospective 3-point scale. The bigger ODI change between the baseline and 3 month follow-up also correlated to better outcome (Figure 3). This data could be used in clinical work to predict possible surgical outcome.

The higher correlation of the 3-point outcome scale with the ODI than with the VAS and BDI is logical. The VAS measured overall back pain, which is, in contrast to neurogenic claudication, usually not the worst symptom relieved by surgery in LSS patients. With regard to the BDI, improvement in disability and pain are the most important aspects of good outcome [4], and depression is only a comorbid condition, although, a potential predictor of outcome. Interestingly, correlations with the VAS and BDI were almost two times higher in patients with only lateral stenosis compared with central stenosis patients. One explanation for this could be that severe lateral spinal stenosis causing nerve compression is the major cause of pain and disability, and patients may have fever other symptomatic structural changes in their spine. One limitation of this study is the relatively small number of patients with lateral spinal stenosis.

## Conclusions

Retrospective assessment of spinal surgery outcome is highly reproducible. Accuracy is highest in the patients with poor and good surgical result.

## Competing interests

The authors report no declarations of interest.

## Authors’ contributions

PK performed the statistical analyses, did the retrospective analysis and drafted the manuscript. TA, TH, SS, HK, VT, OA, VL and PS participated in acquisition of data and helped to draft the manuscript. TA, OA, and HK also participated in the design of the study All authors read and approved the final manuscript.

## Pre-publication history

The pre-publication history for this paper can be accessed here:

http://www.biomedcentral.com/1471-2474/13/83/prepub

## References

[B1] Deyo RA Mirza SK Martin BI Kreuter W Goodman DC Jarvik JG Trends, major medical complications, and charges associated with surgery for lumbar spinal stenosis in older adults JAMA 2010 303 13 1259 1265 10.1001/jama.2010.338 20371784 PMC2885954

[B2] Atlas SJ Keller RB Wu YA Deyo RA Singer DE Long-term outcomes of surgical and nonsurgical management of lumbar spinal stenosis: 8 to 10 year results from the maine lumbar spine study Spine (Phila Pa 1976) 2005 30 8 936 943 10.1097/01.brs.0000158953.57966.c0 15834339

[B3] Birkmeyer NJ Weinstein JN Medical versus surgical treatment for low back pain: evidence and clinical practice Eff Clin Pract 1999 2 5 218 227 10623054

[B4] Haefeli M Elfering A Aebi M Freeman BJ Fritzell P Guimaraes Consciencia J, Lamartina C, Mayer M, Lund T, Boos N: What comprises a good outcome in spinal surgery? A preliminary survey among spine surgeons of the SSE and European spine patients Eur Spine J 2008 17 1 104 116 10.1007/s00586-007-0541-5 17990007 PMC2365536

[B5] Sinikallio S Aalto T Airaksinen O Herno A Kroger H Savolainen S Turunen V Viinamaki H Depression and associated factors in patients with lumbar spinal stenosis Disabil Rehabil 2006 28 7 415 422 10.1080/09638280500192462 16507504

[B6] Sinikallio S Aalto T Airaksinen O Herno A Kroger H Savolainen S Turunen V Viinamaki H Depression is associated with poorer outcome of lumbar spinal stenosis surgery Eur Spine J 2007 16 7 905 912 10.1007/s00586-007-0349-3 17394027 PMC2219645

[B7] Ostelo RW Deyo RA Stratford P Waddell G Croft P Von Korff M Bouter LM de Vet HC Interpreting change scores for pain and functional status in low back pain: towards international consensus regarding minimal important change Spine (Phila Pa 1976) 2008 33 1 90 94 10.1097/BRS.0b013e31815e3a10 18165753

[B8] Fairbank JC Couper J Davies JB O'Brien JP The Oswestry low back pain disability questionnaire Physiotherapy 1980 66 8 271 273 6450426

[B9] Pekkanen L Kautiainen H Ylinen J Salo P Hakkinen A Reliability and Validity Study of the Finnish Version 2.0 of the Oswestry Disability Index Spine (Phila Pa 1976) 2011 36 4 332 338 20823785 10.1097/BRS.0b013e3181cdd702

[B10] Beck AT Ward CH Mendelson M Mock J ERBAUGH J: An inventory for measuring depression Arch Gen Psychiatry 1961 4 561 571 10.1001/archpsyc.1961.01710120031004 13688369

[B11] Raitasalo R Depression and its association with the need for psychotherapy (article in Finnish) 1977 The Social Insurance Institute of Finland publications, Helsinki A 13

[B12] Roland M Morris R A study of the natural history of back pain. Part I: development of a reliable and sensitive measure of disability in low-back pain Spine (Phila Pa 1976) 1983 8 2 141 144 10.1097/00007632-198303000-00004 6222486

[B13] Scott J HE: Graphic representation of pain 1976 2 2 175 184 1026900

[B14] Airaksinen O Herno A Saari T Surgical treatment of lumbar spinal stenosis: patients' postoperative disability and working capacity Eur Spine J 1994 3 5 261 264 10.1007/BF02226576 7866848

[B15] Herno A Airaksinen O Saari T Svomalainen O Pre- and postoperative factors associated with return to work following surgery for lumbar spinal stenosis Am J Ind Med 1996 30 4 473 478 10.1002/(SICI)1097-0274(199610)30:4<473::AID-AJIM13>3.0.CO;2-1 8892553

[B16] Ware JE Sherbourne CD The MOS 36-item short-form health survey (SF-36) I. Conceptual framework and item selection Med Care 1992 30 6 473 483 10.1097/00005650-199206000-00002 1593914

[B17] The EuroQol Group Anonymous EuroQol--a new facility for the measurement of health-related quality of life Health Policy 1990 16 3 199 208 10109801 10.1016/0168-8510(90)90421-9

[B18] Sintonen H The 15D instrument of health-related quality of life: properties and applications Ann Med 2001 33 5 328 336 10.3109/07853890109002086 11491191

[B19] Waddell G Newton M Henderson I Somerville D Main CJ A Fear-Avoidance Beliefs Questionnaire (FABQ) and the role of fear-avoidance beliefs in chronic low back pain and disability Pain 1993 52 2 157 168 10.1016/0304-3959(93)90127-B 8455963

